# *Clostridioides difficile* Infection in Liver Cirrhosis: A Concise Review

**DOI:** 10.1155/2022/4209442

**Published:** 2022-06-07

**Authors:** Yuanbin Liu, Mingkai Chen

**Affiliations:** Department of Gastroenterology, Renmin Hospital of Wuhan University, No. 99 Zhang Zhidong Road, Wuhan 430000, Hubei, China

## Abstract

*Clostridium difficile* is a Gram-positive bacillus with fecal-oral transmission and is currently one of the most common nosocomial infections worldwide, which was renamed *Clostridioides difficile* in 2016. *Clostridioides difficile* infection (CDI) is a prevalent infection in cirrhosis and negatively affects prognosis. This study aimed to provide a concise review with clinical practice implications. The prevalence of CDI in cirrhotic patients increases, while the associated mortality decreases. Multiple groups of risk factors increase the likelihood of CDI in patients with cirrhosis, such as antibiotic use, the severity of cirrhosis, some comorbidities, and demographic aspects. Treatment in the general population is currently described in the latest guidelines. In patients with cirrhosis, rifaximin and lactulose have been shown to reduce CDI risk due to their modulatory effects on the intestinal flora, although conflicting results exist. Fecal microbiota transplantation (FMT) as a treatment for the second or subsequent CDI recurrences has demonstrated a good safety and efficacy in cirrhosis and CDI. Future validation in more prospective studies is needed. Screening of asymptomatic patients appears to be discouraged for the prevention currently, with strict hand hygiene and cleaning of the ward and medical equipment surfaces being the cornerstone of minimizing transmission.

## 1. Introduction

*Clostridium difficile* (*C. difficile*) is a Gram-positive, anaerobic, spore-producing bacillus widespread in the human intestine and the natural environment [1]. In 2016, it was officially renamed *Clostridioides difficile* [2]. Generally, its spores are transmitted by the fecal-oral route and colonize and proliferate in the large intestine [3]. *C. difficile* can release two major protein exotoxins (TcdA and TcdB) that induce colitis in susceptible individuals. However, not all colonized populations are symptomatic [3]. Symptoms evolve from colonization to infection, and colonization by toxigenic strains is an independent risk factor for *Clostridioides difficile* infection (CDI) [4]. *C. difficile* was first isolated in the stool of a newborn in 1935 [5], and until the 1970s, this group was perceived to be symbiotically related to humans [3, 5]. Following the introduction of antibiotics, the incidence of CDI has escalated and now constitutes one of the most common nosocomial infection pathogens [3]. As per a 2015 United States (US) survey, CDI is the most common healthcare-associated infection in the US, standing at approximately 15% [6]. In a recent extensive systematic review [7], the overall incidence of CDI in European countries varied from the lowest in Spain (2.33 per 10,000 patient days) to the highest in Poland (7.88 per 10,000 patient days). The incidence of CDI overall was 53.5 cases per 100,000 adults in 2019 in a recent epidemiological survey in Hong Kong, China [8]. In an analysis of health system data conducted in the US, CDI hospital management required nearly 2.4 million days of hospital stays in the ten years from 2005 to 2015, which imposed a substantial financial burden on the country [9]. Of note is that community-based CDI is incrementally on the rise, further exacerbating the disease burden associated with CDI [10]. Antibiotic use [11], old age [12], gastric acid inhibitors [13], and hospitalization [14] are the critical risk factors for the development of such infections.

Cirrhosis is the end stage of chronic liver disease and is responsible for a heavy burden of illness and death worldwide. In 2017, cirrhosis caused more than one million deaths [15, 16], making it the eleventh leading cause of death [15]. Data from 2019 showed that cirrhosis contributed to 560.4 age-standardized disability-adjusted life years (DALYs) per 100,000 population (one DALY represents one life-year of full health lost) [17]. Infection is a significant comorbidity in patients with cirrhosis, increasing mortality risk [18–20]. Since risk factors for the development of CDI are also frequently reported in patients with cirrhosis [21], CDI is also a prevalent type of infection in cirrhosis and hurts the prognosis of patients. Cirrhosis with CDI have a worse prognosis and more extended hospital stays than those without CDI [22]. Meanwhile, the incidence of CDI is double that of noncirrhotic patients, and there are more CDI-related complications compared with patients without cirrhosis [23]. Recurrent CDI (R-CDI) disease burden in cirrhotic patients is even more challenging [22]. Fecal microbiota transplantation (FMT) is the recommended treatment for R-CDI [24]. Still, its implementation in patients with cirrhosis is questionable [25, 26] as it raises the possibility of additional adverse events in decompensated cirrhosis [25]. Other relevant therapies such as rifaximin [27] and lactulose [28] have also shown evidence in reducing CDI. Rational understanding of the impact of CDI in cirrhosis and treatment options to improve outcomes and lower the burden of disease on patients is therefore highly regarded.

Given the magnitude of the disease burden posed by CDI in cirrhosis and the controversial nature of some of the issues, therefore, this study aims to provide clinicians with a synthesis of the latest status on the epidemiology, risk factors, prognosis, and therapeutic aspects of CDI in patients with cirrhosis and briefly characterize the impact of cirrhosis in CDI hospitalization. As the pathogenesis in cirrhosis, clinical presentation, and diagnosis of CDI have been well described [28, 29], these sections will not be discussed in this review.

## 2. Method

The electronic databases PubMed and Embase were retrieved manually to obtain relevant literature. The reference lists in the primary included literature were also checked internally to search for matches. Only publications in the English language were included. There was no restriction on the year of publication for the documents. We excluded studies that primarily involved patients receiving liver transplants, as the profile of CDI in this specific population is somewhat different from that of the general cirrhotic population. Studies that included populations younger than 18 years were excluded. Index terms included “cirrhosis,” “*Clostridium difficile*,” “*Clostridioides difficile,*” “*Clostridium difficile* infection,” “chronic liver disease,” and “infection.” A critical evaluation was carried out for all studies included in this paper.

## 3. Epidemiology

Several studies have reported the prevalence of CDI in patients with cirrhosis using large nationwide databases. In a study conducted in an extensive commercial database in the US, the prevalence of CDI in cirrhosis was 134.93 per 100,000 of 133,400 patients diagnosed with cirrhosis between 2018–2021 [30]. Nationwide Readmissions Database (NRD) in the US revealed that the prevalence of CDI in patients with cirrhosis was 2.8% from 2011 to 2014, with higher inpatient mortality compared with cellulitis and urinary tract infections (UTI) (17.6% vs. 7.6%, 11.8%), respectively, and the presence of sepsis and organ failure was also most common in CDI [31]. Another study using the National Inpatient Sample (NIS) [32], which investigated trends in CDI hospitalizations for end-stage liver disease (ESLD) from 2005 to 2014, found that the prevalence of CDI among inpatients with decompensated cirrhosis increased approximately twofold from 1.3% in 2005 to 2.7% in 2014, with an annual rate of increase of 7.8%. However, mortality in patients with in-hospital ESLD including CDI decreased notably from 15.4% in 2005 to 11.1% in 2015, a decrease that improved diagnostic and therapeutic approaches can explain. Similar results were observed in several other studies that used NIS in patients with advanced cirrhosis to describe CDI prevalence and mortality [23, 33, 34].

Some local data also provide epidemiological figures for CDI in cirrhosis. In a study from a tertiary hospital in Romania [35], CDI occurred in 7.3% of 231 patients with cirrhosis coexisting with hepatic encephalopathy (HE) (mainly stage 2 or 3) between 2012 and 2014, with an overall CDI incidence of 57.2 cases per 10,000 patient days. In a small prospective study conducted in Romania in 2015, among 200 Child-Pugh B and C patients hospitalized for decompensation, 9% developed CDI during their hospitalization [36]. Another prospective study, also conducted in a Romanian tertiary hospital, included 122 patients with cirrhosis and spontaneous bacterial peritonitis (SBP) who also received norfloxacin as secondary prophylaxis from 2018 to 2019, in which 18.8% of the population presented with CDI (median follow-up of 7 months) [37]. In a further over six years study, CDI incidence was 11.8% in 388 cirrhotic patients, and notably, 30.8% of the cirrhotic patients received the antibiotic rifaximin to prevent HE [38]. In a study of patients with variceal bleeding, also conducted in Romania, the incidence of CDI was 6.8% between 2017 and 2019 [39]. Finally, in another hospital in China, the Infectious Diseases Department reported 26 cases of CDI in 526 cirrhotic inpatients over six months in 2015 (4.9%) [4].

The incidence of R-CDI in patients with cirrhosis was studied in a cohort study conducted at Indiana University Hospital from 2012 to 2016, with an 11.9% incidence of R-CDI among those hospitalized with CDI in patients with cirrhosis [21].

The prevalence of cirrhosis among 366,283 inpatients with CDI between 2011 and 2014 was 3.4%, according to the survey conducted in the NRD [40]. Of these cirrhotic patients, 63.1% had decompensated cirrhosis. Another two studies using NIS yielded a 3.97% and 4.18% prevalence of cirrhosis in 2012–2015 and 2016-2017, respectively [41, 42]. A further study implemented in a US health system diagnosed cirrhosis in 9.13% of 526 CDI inpatients from 2014 to 2017 [43]. However, an additional 2011 study based on long-term care facilities (LTCFs) showed only 326 (0.72%) cirrhotic patients out of 45,500 CDI admissions [44]. This is presumably explained by the fact that the number of CDI admissions rather than the specific number of people was considered (some patients had readmissions), and the database only included individuals ≥65 years (median age 82 years), which resulted in a significantly higher prevalence of CDI.

In summary, these nationwide population studies in the US demonstrate an overall increasing trend in CDI prevalence in patients with cirrhosis, in contrast to decreasing associated mortality. CDI incidence in local hospitals reported in the literature is even higher. The incidence of R-CDI in cirrhosis is not uncommon. Cirrhosis accounts for approximately 3-4% of CDI hospitalizations in nationwide studies. However, data from other parts of the world are still lacking (Table 1).

## 4. Risk Factors

**Figure 1:**
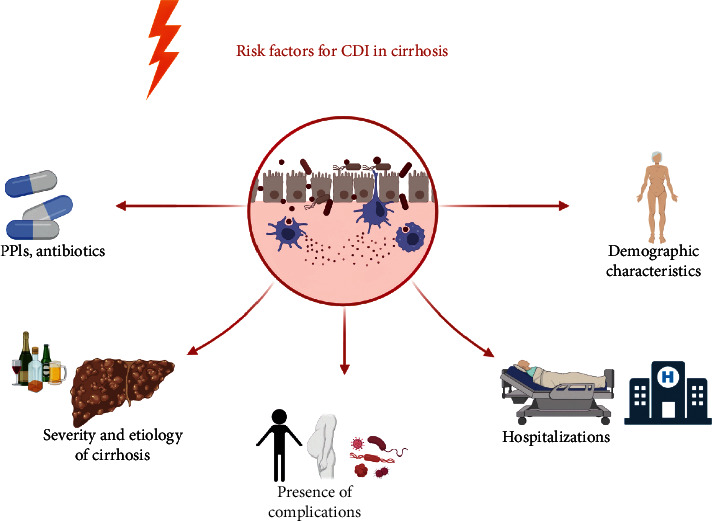
Risk factors for CDI development in patients with cirrhosis. The risk factors for the development of CDI in patients with cirrhosis have been described in different studies. In general, they can be divided into several categories: namely medications (PPIs, antibiotics, etc.), severity and etiology of cirrhosis (Child-Pugh grade, Charlson index, alcoholic etiology, etc.), presence of complications (HE, hypoproteinemia/malnutrition, infections, hepatorenal syndrome, ascites, etc.), hospitalizations (multiple hospitalizations, extended hospital stays, ICU admissions), demographic characteristics (advanced age, female, ethnicity) and CDC. Abbreviations: CDI, *Clostridioides difficile* infection; PPIs, proton pump inhibitors; HE, hepatic encephalopathy; ICU, intensive care unit; CDC, *Clostridioides difficile* colonization.

The risk factors for the development and progression of cirrhosis have been well established. Age >65 years, multiple hospitalizations, inpatient stays >20 days, hypoproteinemia, *Clostridioides difficile* colonization (CDC), HE, antibiotic, and proton pump inhibitors (PPIs) use were found to be associated with the development of CDI in a study conducted to identify risk factors for CDI in patients with cirrhosis [45]. Furthermore, many studies have also reported risk factors for CDI development [22, 23, 30, 32, 35, 37, 39, 46–49] although heterogeneity exists between studies. The risk factors concluded from these studies are largely in line with the previous research and can be broadly classified into several categories, namely medications (PPIs, antibiotics, etc.), severity and etiology of cirrhosis (Child-Pugh grade, Charlson index, etc.), presence of complications (HE, hypoproteinemia/malnutrition, infections, hepatorenal syndrome, ascites, etc.), hospitalizations (multiple hospitalizations, extended hospital stays, etc.), demographic characteristics of the patients (advanced age, female, ethnicity), and CDC. Several issues require further clarification in this regard. Firstly, studies have shown females to be more prone to CDI [50]. This was confirmed in a couple of studies on cirrhosis patients [22, 30, 32, 38, 47]. Experimental and human studies have demonstrated differences in the gut microbiome concerning gender, and such effects are mediated by sex hormone levels [51, 52]. However, studies on the sex differences in *C. difficile* abundance have not yet emerged. Secondly, etiological variants in cirrhosis may also be a risk factor for CDI. Nonalcoholic fatty liver disease (NAFLD) is associated with an increased risk of CDI [53] although no studies have shown that this etiology increases CDI risk in cirrhosis. A few studies suggest that alcoholic etiology is a risk factor for CDI [37, 47]. Lastly, medication use such as rifaximin and PPIs shows conflicting results in this context. Several studies have shown rifaximin to be protective and therapeutic (as mentioned later) [27, 54–56]. PPIs are risk factors for CDI in many studies, but PPIs were not shown to cause CDI in an evidence-based review, although there may be an increased risk of infectious diarrhea [57]. In other words, no substantial evidence is available for a causative relationship for PPIs on CDI in the general population and the cirrhotic population although an increased risk is identified. The impact of these agents on CDI in cirrhosis needs to be further supported in high-quality studies. A study showed risk factors for R-CDI in cirrhosis, including Charlson Comorbidity Index and lactulose use, which is aligned with the risk factors for CDI [21] (Figure 1).

Overall, the risk factors for the development of CDI in cirrhosis fall into several broad categories, that is, certain established drug exposures, progression of cirrhosis and specific etiology, presence of complications, hospitalization, patient demographic characteristics, and CDC, of which several still warrant further exploration. Being aware of the predisposing factors for the occurrence of CDI in patients with cirrhosis has positive implications for timely insight and subsequent prevention and treatment by clinicians.

## 5. Prognosis

Increased mortality and comorbidity are associated with infection in cirrhosis. Given the dramatic advances in healthcare management, in-hospital mortality in cirrhotic patients has declined [58], and mortality in CDI patients has also been dropping annually. However, its associated mortality and burden of complications remain significantly overwhelming compared with other populations, increasing mortality by about 50% in patients with cirrhosis and CDI versus those without CDI [33]. Therefore, understanding and predicting the prognosis of this population is essential to mitigate the risk of undesirable outcomes. Extensive publications have reported increased mortality of CDI in patients with cirrhosis [22, 23, 31, 33, 34, 36, 39, 44, 47, 48, 59]. However, one study has not established the impact of CDI development on mortality in patients with cirrhosis and SBP receiving norfloxacin as secondary prophylaxis [37]. Norfloxacin has been shown in in vitro studies as a quinolone to down-regulate inflammation, which may be a protective effect [60, 61]. Yet another study indicated that CDI was associated with increased 30-day mortality but not with increased overall mortality [48]. Alongside increased mortality, CDI could potentially carry an additional risk of complications, including sepsis [31], organ failure [31], portal vein thrombosis [62], and readmission [21]. Caution should be taken, as readmission is associated with increased severity of cirrhosis and mortality [21, 40]. Studies on the outcomes of CDI in cirrhosis are summarized in Table 2.

The impact of cirrhosis on inpatients with CDI has been addressed in several studies. In a retrospective study using the NIS database between 2012 and 2015, the presence of cirrhosis in CDI admissions was associated with increased mortality, with an adjusted hazard ratio (aOR) of 1.65 and a 95% confidence interval (CI) of 1.53–1.77 [41]. Another study revealed similar results using the NRD between 2011 and 2014 [40]. A further study conducted in 526 CDI admissions found a significantly higher mortality among the cirrhotic population than the noncirrhotic group (39.6% vs. 14.6%, *p*=0.001) [43]. An association was also established with the presence of cirrhosis and 30-day readmission for CDI [63]. Nonetheless, a study using data from the NIS during 2016-2017 found that cirrhosis was not associated with increased all-cause mortality (aOR 1.31, 95% CI 0.89–1.93) [42]; this may represent a change that has evolved in more recent years.

CDI is an independent predictor of mortality in patients with cirrhosis [32, 33, 64]. Predicting mortality in patients with cirrhosis and CDI for targeted intervention is thus crucial. The model for end-stage liver disease (MELD) was identified as the only predictor of 30-day mortality in one study [59]. A second study suggested that hypoalbuminemia (albumin <3 g/dL) and intensive care unit (ICU) admission were independent predictors of short-term mortality [48]. A consideration of the discrepancy may arise from differences in the measured outcomes in the two studies. The outcomes in the study that yielded MELD as the sole predictor were 30-day mortality, 30-day colectomy, any requirement for ICU admission, and R-CDI within 90 days, whereas in the latter, the primary outcomes were 30-day mortality and overall mortality. ICU admission was adopted as an outcome instead of a prognostic indicator in the first study. The further point is that the latter excluded the MELD score in the multivariate analysis. Therefore, the inclusion of MELD in the multivariate analysis allowed for consideration of the severity of cirrhosis, detracting from the prognostic value of hypoalbuminemia [59]. To conclude, in a broad sense, both suggest that the severity of cirrhosis is a predictor of death among the CDI population in cirrhosis.

## 6. Treatment

### 6.1. General Considerations

Recently, two American guidelines have described the treatment options for CDI in the general population [65, 66]. For the initial episode of nonsevere CDI, oral vancomycin 125 mg 4 times daily for ten days or oral fidaxomicin 200 mg twice daily for ten days is recommended [65]. In contrast, fidaxomicin is superior to vancomycin for the Infectious Diseases Society of America (IDSA) and Society for Healthcare Epidemiology of America (SHEA) guidelines [66]. Oral metronidazole 500 mg 3 times daily for ten days may be an alternative selection if the above two first-line agents are not available or in a low-risk CDI population. However, initial therapy for severe CDI remains with two first-line drugs at the same dose and for the same duration. As initial treatment for fulminant CDI, oral vancomycin 500 mg 4 times daily in combination with parenteral metronidazole 500 mg every 8 hours is recommended. An additional vancomycin enema of 500 mg can be administered every 6 hours if ileus is present. Sufficient capacity must also be available for resuscitation [65]. For the first recurrence of CDI, a tapering/pulsed dose of vancomycin is recommended (if the standard regimen was used for the initial episode). If the initial treatment is given with metronidazole or vancomycin, fidaxomicin is recommended [65]. In the IDSA and SHEA guidelines, fidaxomicin is preferred to vancomycin, and bezlotoxumab (a human monoclonal antibody against C. difficile toxin B) 10 mg/kg given intravenously is also recommended as adjunctive therapy to antibiotic therapy [66]. The notable difference in the two guidelines for the second or subsequent recurrence of CDI is that the American College of Gastroenterology (ACG) guidelines recommend FMT for this population [65]. In contrast, the IDSA and SHEA guidelines suggest that FMT be performed after at least two recurrences treated with antibiotics [66] (Table 3).

In addition, the latest guideline from the European Society of Clinical Microbiology and Infectious Diseases (ESCMID) [67] is also available. In the ESCMID guideline, the standard of care (SOC) for initial CDI is fidaxomicin 200 mg twice daily for ten days or vancomycin 125 mg 4 times daily for ten days, and for those at high risk of recurrence, fidaxomicin or SOC plus bezlotoxumab is considered. If the preferred option is not available, metronidazole 500 mg 3 times daily for ten days is recommended. For the first recurrence, SOC plus bezlotoxumab or fidaxomicin is recommended, while FMT or SOC plus bezlotoxumab is recommended for a second recurrence, and if it fails, vancomycin is used by tapering and pulsed. The guideline refers to severe CDI as a separate clinical type, regardless of the number of previous episodes.

### 6.2. Rifaximin

Rifaximin, a derivative of rifamycin, is poorly absorbed in the gut and is currently prescribed as a therapeutic agent for recurrent HE and exerts its antibacterial activity by inhibiting RNA synthesis in bacteria [68, 69]. Rifaximin is a therapeutic for CDI [70–72]. In the latest guideline [66], rifaximin 400 mg 3 times daily for 20 days as continuation therapy to vancomycin can be offered as a treatment for the second or subsequent recurrences of CDI. There are some conflicting effects of rifaximin on CDI in cirrhosis. A few studies suggest that rifaximin is a risk factor for developing CDI in patients with cirrhosis [47, 49]. A few reasons may explain in these studies that rifaximin may have increased CDI risk in patients with cirrhosis. First, in the survey by Bajaj et al. [47], the risk of nosocomial infection was increased in a regression model including rifaximin use, but the model was not robust enough, and rifaximin was used as a surrogate for HE as a variable in this study, and HE is a known risk factor for the development of CDI. Secondly, in another Spanish study including 46 patients with cirrhosis and CDI, 34.1% were rifampin-resistant strains, and 84.6% were in patients who had previously received rifaximin [38]. This is in line with the study also conducted in Spain that reported an increased risk of CDI due to rifaximin, which also reported a high incidence of rifaximin-resistant strains [49]. Rifaximin-resistant strains were significantly more often female, had a higher incidence of HE and portal hypertension, and were more frequently treated with rifaximin or rifamycin [38], which may contribute to the increased incidence of CDI. Apart from these local data, rifaximin was shown to reduce CDI development while treating HE [27, 59, 73], and rifaximin also showed no increase in rifaximin-resistant strains during the treatment of HE in a systematic review and meta-analysis [74]. Thus, alongside HE treatment, rifaximin has shown a more positive effect on CDI in cirrhosis, and yet further prospective studies are needed.

### 6.3. Lactulose

Lactulose is a nondigestible oligosaccharide frequently combined with rifaximin as prevention for HE [75]. It promotes the growth of indigenous host microorganisms as a prebiotic and enhances colonization resistance to CDI [76, 77]. Similarly, lactulose can be used as a substitute for HE or the severity of cirrhosis and is, therefore, a risk factor for developing CDI [47] and R-CDI [21] in some studies. A case-control study revealed a significantly lower incidence of CDI with the combination of lactulose and rifaximin compared with lactulose alone (12.5% vs. 27.9%, *p*=0.02). A nested controlled study confirmed the positive effect of lactulose on CDI [78], including 112 patients with decompensated cirrhosis and incident CDI and 928 matched controls, and lactulose significantly reduced the incidence of CDI after excluding patients who received rifaximin (aOR 0.52, 95% CI 0.31–0.89, *p*=0.02). Controversy remains regarding the use of lactulose in patients with cirrhosis to reduce CDI risk concurrently, and prospective studies are awaited to elucidate the issue further.

### 6.4. Fecal Microbiota Transplantation

FMT has demonstrated superior efficacy in recurrent CDI as solid evidence of the role of microbiota in the diseases [79]. Since FMT was first recommended in guidelines in 2013 as a treatment for the third recurrence of CDI [80], it has been officially endorsed for its role in the treatment of recurrent CDI and the latest guidelines [65, 66], and as mentioned above, FMT is recommended as the treatment for second or further recurrences of CDI. However, the administration of FMT in patients with cirrhosis and CDI has not been much specified. In an FMT Working Group review in 2011 [25], decompensated cirrhosis and other forms of severe immunodeficiency were regarded as conditions that would lead to increased risk of adverse events with FMT and were not recommended for implementation. Recently, however, the use of FMT has appeared to gain more clarity regarding its safety and efficacy in patients with cirrhosis and even decompensated cirrhosis. The trial of FMT outcomes in recurrent HE demonstrated a favorable effect on hospitalization, cognitive improvement, and dysbiosis in patients with cirrhosis [81]. Similar findings were obtained for long-term FMT with a high safety profile [82]. Based on these encouraging results, positive effects were noted in patients with cirrhosis and recurrent CDI. A retrospective study included 63 patients with cirrhosis (median MELD, 14.5; 24 patients with decompensated cirrhosis) undergoing FMT in multiple centers from 2012–2018, yielding a final FMT success of 85.7%, with adverse events (AEs) and serious adverse events (SAEs) occurring in 21 and 5 patients, respectively [26]. The AEs that may be linked to FMT consisted of abdominal pain/cramping and diarrhea. The occurrence of SAEs was rare, and the five cases included hospitalization associated with a Crohn's disease flare, fecal urgency, dehydration due to acute kidney injury, and cirrhotic decompensation possibly involved with FMT. Efficacy and safety of FMT in patients with cirrhosis and CDI were demonstrated, notwithstanding adverse events. Although FMT has shown a positive effect on CDI in the cirrhotic population, more well-designed studies are warranted for closer validation, and meticulous follow-up is still essential to systematically monitor the emergence of complications in clinical practice [83].

### 6.5. Summary

CDI treatment in the cirrhotic population currently has general considerations and some specific possible alternatives. Recently, two American guidelines, a European guideline, and a Taiwanese guideline have recommended CDI treatment, and there are discrepancies between these guidelines. A potential therapeutic effect on the reduced incidence of CDI has been shown in several studies by the two agents for HE prevention, rifaximin and lactulose. However, controversial results remain, and more large sample studies are needed to demonstrate the issue in the future. FMT has shown promising safety and efficacy in patients with cirrhosis and CDI.

## 7. Prevention

Prevention of the development of CDI in patients with cirrhosis necessitates several interventions. The first is the introduction of potentially appropriate screening strategies, and the second is minimizing identified and controllable risk factors. Finally, emphasis should be placed on hand hygiene and the decontamination of medical equipment and wards.

Saab and colleagues present two strategies for screening and treating CDI [84]. The first strategy involved screening all cirrhotic patients and treating those who were positive instead of treating only individuals with symptomatic CDI without screening. A Markov model was developed to compare the respective healthcare costs and patient outcomes between the proposed strategies. Screening for CDI in all populations showed a 3.54-fold reduction in associated medical costs and lower mortality among patients with symptomatic CDI. This study demonstrated that screening and treating asymptomatic patients were cost-effective and prevented more complications than not screening. However, this contradicted the available clinical guidelines [65]. The guidelines recommended only testing for *C. difficile* in the diarrheal stools and discouraged treatment of *C. difficile* carriers. Additional concerns from other authors have prompted discussions on CDI screening [36, 85]. Zacharioudakis et al. provided a systematic review and meta-analysis of the prevalence of toxicogenic CDC and the risks of infection in hospitalized populations, and the prevalence of colonization in the asymptomatic people was found to be 8.1%, with a significantly higher risk of developing CDI (21.8% vs. 3.4%) [86]. However, only 154 patients (1.8%) were screened for CDI in this analysis, including 8725 inpatients [85]. As most patients screened would not progress to CDI, it might seem impractical to screen asymptomatic populations. Furthermore, asymptomatic patients are a source of *C. difficile* transmission in the general population. In cirrhosis, the risk of transmission should be increased due to impaired immunity and complications. Given the widespread availability of disinfection measures today, e.g., hand washing, however, the potential for transmission between these asymptomatic patients would be limited [85]. A separate study found that CDI developed after antibiotic therapy in 200 patients with cirrhosis and identified multiple antibiotic therapies as the only independent risk factor. Therefore, Pop et al. indicated that screening for CDI in the asymptomatic population should only be implemented if the cirrhotic population is at high risk for CDI [36].

Several studies have demonstrated that screening asymptomatic hospitalized populations can reduce the incidence of nosocomial CDI and may be recommended for clinical practice [87–91]. Nevertheless, the models in these studies were established in the general hospitalizations with no further evidence of generalization in the distinct subpopulation of cirrhosis. More research is needed to support CDI screening in an asymptomatic patient with cirrhosis. Testing of symptomatic patients in the cirrhotic population should currently be mandatory.

Infection control-based approaches (antibiotic stewardship, improved hygiene concepts to reduce transmission within the ward) remain the cornerstone of the prevention of hospitalized CDI patients. Strict disinfection routines, including cleaning stethoscopes and other medical equipment, and thorough sterilization of wards to eliminate possible residual spores on surfaces, are fundamental to prevent transmission [85]. These basic precautions are even further emphasized in patients with cirrhosis. The prevention and treatment of hypoalbuminemia are of clinical relevance in preventing infections [92], notably CDI in patients with cirrhosis [93], and serum levels of the effective albumin may be more significant [94]. Antibiotics and PPIs administered for complications are frequently used in patients with cirrhosis; the judicious use of broad-spectrum antibiotics and PPIs is probably supportive in preventing CDI.

## 8. Conclusion

Currently known as one of the most common nosocomial infections, CDI is as well a common type of infection in the cirrhotic population. Nationwide databases have shown that the prevalence of CDI in cirrhosis has been on the rise in recent years, while the associated mortality has been falling. Large databases indicate that cirrhosis comprises approximately 3-4% of CDI admissions, with local hospital data varying considerably. CDI imposes a heavy economic burden on cirrhosis, carrying higher mortality and the development of complications. The severity of cirrhosis may be a predictor of death from CDI. Numerous factors may contribute to the susceptibility of individuals with cirrhosis to CDI, such as antibiotics and PPIs and severity and complications of cirrhosis and hospitalization. Appropriate prevention and treatment are crucial to reduce the disease burden of CDI in the cirrhotic population. The treatment of CDI is specified in the latest guidelines. In the setting of cirrhosis, agents such as rifaximin and lactulose used to prevent recurrent HE have shown controversial results. FMT as a preferred option for the treatment of second or subsequent recurrent CDI has also demonstrated a promising safety and efficacy profile in cirrhosis and CDI, but careful follow-up is still necessary. Screening of asymptomatic populations currently appears not to be recommended, but thorough disinfection of wards and medical equipment remains the cornerstone of preventing CDI transmission.

## Figures and Tables

**Table 1:** Epidemiological profile related to CDI and cirrhosis. Abbreviations: CDI, *Clostridioides difficile* infection; US, United States; SNOMED–CT, systematized nomenclature of medicine clinical terms; NRD, nationwide readmissions database; NIS, national inpatient sample; ICD, international classification of diseases; CLD, chronic liver disease; HE, hepatic encephalopathy; EIA, enzyme immunoassay; SBP, spontaneous bacterial peritonitis; GDH, glutamate dehydrogenase; PCR, polymerase chain reaction; LTCFs, long-term care facilities.

Reference	Country	Study type	Study duration	Patient cohort	Database	CDI diagnostic methods	Epidemiology
*CDI in cirrhosis*							
[30]	US	Retrospective	2018–2021	133,400 patients with cirrhosis	Explorys	SNOMED-CT	Prevalence: 134.93 per 100,000
[31]	US	Retrospective	2011–2014	1,798,830 patients with cirrhosis	NRD	ICD-9	Prevalence: 2.8%
[32]	US	Retrospective	2005–2014	590,980 patients with decompensated cirrhosis	NIS	ICD-9	Prevalence: from 1.3% in 2005 to 2.7% in 2014; in-hospital mortality: from 15.4% in 2005 to 11.1% in 2014
[33]	US	Retrospective	1998–2014	3, 049, 696 patients with advanced cirrhosis	NIS	ICD-9	Prevalence: from 0.8% in 1998 to 2.6% in 2014; in-hospital mortality: from 20.7% in 1998 to 11.3% in 2014
[34]	US	Retrospective	1998–2007	742, 391 patients with cirrhosis	NIS	ICD-9	Prevalence: from 0.7% in 1998 to 1.6% in 2007; in-hospital mortality: from 13.4% in 1998 to 12.3% in 2007
[23]	US	Retrospective	2009	114,108 patients with CLD	NIS	ICD-9	Incidence: 189.4/10,000
[35]	Romania	Retrospective	2012–2014	231 patients with cirrhosis and HE	Tertiary hospital	EIA	Incidence: 7.3%
[36]	Romania	Prospective	2015	200 patients with decompensated cirrhosis	Tertiary hospital	EIA	Incidence: 9%
[37]	Romania	Prospective	2018–2019	122 patients with cirrhosis and SBP receiving secondary prophylaxis with norfloxacin	Tertiary hospital	EIA	Incidence: 18.8%
[38]	Spain	Retrospective	2009–2014	367 patients with cirrhosis and 30.8% received rifaximin	Tertiary hospital	Rapid detection test	Incidence: 11.8%
[39]	Romania	Retrospective	2017–2019	367 patients with cirrhosis and variceal bleeding	Tertiary hospital	EIA	Incidence: 6.8%
[4]	China	Retrospective	2015	526 patients with cirrhosis	Tertiary hospital	EIA	Incidence: 4.9%

*R-CDI in cirrhosis*							
[21]	US	Retrospective	2012–2016	257 patients with cirrhosis and CDI	Tertiary hospital	EIA	Incidence: 11.9%

*Cirrhosis in CDI*							
[40]	US	Retrospective	2011–2014	366,283 CDI inpatients	NRD	ICD-9	Prevalence: 3.4%
[41]	US	Retrospective	2012–2015	1,327,595 CDI inpatients	NIS	ICD-9	Prevalence: 3.97%
[42]	US	Retrospective	2016–2017	196,945 CDI inpatients	NIS	ICD-9	Prevalence: 4.18%
[43]	US	Retrospective	2014–2017	526 CDI inpatients	Tertiary hospital	GDH and PCR	Prevalence: 9.13%
[44]	US	Retrospective	2011	45,500 CDI inpatients	LTCFs	ICD-9	Prevalence: 0.72%

**Table 2:** Studies reporting the effect size of the outcomes of CDI in cirrhosis. Abbreviations: CDI, *Clostridioides difficile* infection; US, United States; NIS, national inpatient sample; 95%CI, 95% confidence interval; OR, odds ratio; aOR, adjusted odds ratio; NA, not available; HE, hepatic encephalopathy; SBP, spontaneous bacterial peritonitis; LTCFs, long-term care facilities.

Reference	Study period	Country	Database	Outcome metrics	Effect size (95%CI)	Adjustment factors
[22]	2015	US	NIS	Mortality	aOR: 1.55 (1.29–1.85)	Hospital location, teaching status, insurance status, complications of cirrhosis and infections
[23]	2009	US	NIS	Mortality	aOR: 2.29 (1.90–2.76)	Demographic (age in decade-long intervals, gender, race) and socioeconomic characteristics (primary payer and income level)
[31]	2011–2014	US	NRD	Mortality; sepsis; any organ failure; 2+ organ failures; 30-day readmission	OR:2.00 (1.91–2.28); 3.99 (3.86–4.12); 3.00 (2.90–3.11); 3.25 (3.12–3.39); 1.01 (0.95–1.06), respectively	NA
[33]	1998–2014	US	NIS	Mortality	aOR: 1.47 (1.40–1.56)	Age >65, gender, HE, SBP, variceal bleed, presence of ascites, and Elixhauser comorbidity index
[44]	2011	US	LTCFs	Mortality	aOR: 1.27 (1.24–1.30)	NA

**Table 3:** Recommendations of the latest two American guidelines on the therapeutic aspects of CDI. Abbreviations: ACG, American college of gastroenterology; IDSA, infectious diseases society of America; SHEA, society for healthcare epidemiology of America; CDI, *Clostridioides difficile* infection; NA, not available; FMT, fecal microbiota transplantation; SOC, standard of care.

Clinical definition	ACG guideline [65]		IDSA and SHEA guideline [66]	
	Recommendations	Strength of recommendation, quality of evidence	Recommendations	Strength of recommendation, quality of evidence
Initial episode of nonsevere CDI	Oral vancomycin 125 mg 4 times daily for ten days; oral fidaxomicin 200 mg twice daily for ten days; oral metronidazole 500 mg 3 times daily for ten days in low-risk patients	Strong recommendation, low quality of evidence; strong recommendation, moderate quality of evidence; strong recommendation, moderate quality of evidence, respectively	Preferred: Fidaxomicin 200 mg given twice daily for ten days; Alternative: Vancomycin 125 mg given four times daily by mouth for ten days; if above agents are unavailable: Metronidazole, 500 mg 3 times daily by mouth for 10–14 days	Conditional recommendation, moderate certainty of evidence
Initial episode of severe CDI	Vancomycin 125 mg 4 times a day for ten days; fidaxomicin 200 mg twice daily for ten days	Strong recommendation, low quality of evidence; conditional recommendation, very low quality of evidence, respectively	Preferred: Fidaxomicin 200 mg given twice daily for ten days; Alternative: Vancomycin 125 mg given four times daily by mouth for ten days	Conditional recommendation, moderate certainty of evidence
Fulminant CDI	Adequate volume resuscitation and 500 mg of oral vancomycin every 6 hours daily for the first 48–72 hours; combination therapy with parenteral metronidazole 500 mg every 8 hours; addition of vancomycin enemas 500 mg every 6 hours if ileus; FMT for severe and fulminant CDI refractory to antibiotic therapy	Strong recommendation, very low quality of evidence; conditional recommendation, very low quality of evidence; conditional recommendation, very low quality of evidence; strong recommendation, low quality of evidence, respectively	Vancomycin 500 mg 4 times daily by mouth or by nasogastric tube and intravenously administered metronidazole 500 mg every 8 hours; rectal instillation of vancomycin if ileus	NA
First CDI recurrence	Tapering/pulsed dose vancomycin for a first recurrence after an initial course of fidaxomicin, vancomycin, or metronidazole; fidaxomicin for a first recurrence after an initial course of vancomycin or metronidazole	Strong recommendation, very low quality of evidence; conditional recommendation, moderate quality of evidence, respectively	Preferred: Fidaxomicin 200 mg given twice daily for ten days or twice daily for five days followed by once every other day for 20 days; Alternative: Vancomycin by mouth in a tapered and pulsed regimen or 125 mg given four times daily for ten days; Adjunctive treatment: Bezlotoxumab 10 mg/kg given intravenously once during the administration of SOC antibiotics	Conditional recommendation, low certainty evidence^a^
Second or subsequent CDI recurrence	FMT delivered through colonoscopy or capsules; by enema, if other methods are unavailable; repeat FMT for a recurrence of CDI within eight weeks of an initial FMT; suppressive oral vancomycin for not candidates for FMT, relapsed after FMT, or require ongoing or frequent courses of antibiotics	Strong recommendation, moderate quality of evidence; conditional recommendation, low quality of evidence; conditional recommendation, very low quality of evidence; conditional recommendation, very low quality of evidence, respectively	Fidaxomicin 200 mg given twice daily for ten days or twice daily for five days followed by once every other day for 20 days; vancomycin by mouth in a tapered and pulsed regimen or 125 mg 4 times daily for ten days followed by rifaximin 400 mg 3 times daily for 20 days; FMT after antibiotic treatments for at least two recurrences; Adjunctive treatment: Bezlotoxumab 10 mg/kg given intravenously once during the administration of SOC antibiotics	Conditional recommendation, very low certainty of evidence^b^

^a^fidaxomicin rather than vancomycin. ^b^bezlotoxumab as a co-intervention along with SOC antibiotics rather than SOC antibiotics alone.

## Data Availability

All data are presented in the article, and no additional data are available.
